# Active Surveillance in Non-Muscle Invasive Bladder Cancer, the Potential Role of Biomarkers: A Systematic Review

**DOI:** 10.3390/curroncol31040163

**Published:** 2024-04-12

**Authors:** Diego Parrao, Nemecio Lizana, Catalina Saavedra, Matías Larrañaga, Carolina B. Lindsay, Ignacio F. San Francisco, Juan Cristóbal Bravo

**Affiliations:** 1 School of Medicine, University of O’Higgins, Rancagua 282000, Chile; diego.parrao@pregrado.uoh.cl (D.P.); nemecio.lizana@pregrado.uoh.cl (N.L.); catalina.saavedra@pregrado.uoh.cl (C.S.); 2 Department of Urology, Libertador Bernardo O’Higgins Regional Hospital, Rancagua 282000, Chile; mlarranagar@uft.edu; 3 Research Department, Libertador Bernardo O’Higgins Regional Hospital, Rancagua 282000, Chile; carolina.lindsay@ug.uchile.cl; 4 Division of Urologic Surgery, Beth Israel Deaconess Medical Center, Harvard Medical School, Boston, MA 02215, USA; isanfran@bidmc.harvard.edu

**Keywords:** bladder cancer, NMIBC, active surveillance, biomarkers, TURBT, cystoscopy

## Abstract

Bladder cancer (BC) is the tenth most common cause of cancer worldwide and is the thirteenth leading cause of cancer mortality. The non-muscle invasive (NMI) variant represents 75% of cases and has a mortality rate of less than 1%; however, it has a high recurrence rate. The gold standard of management is transurethral resection in the case of new lesions. However, this is associated with significant morbidity and costs, so the reduction of these procedures would contribute to reducing complications, morbidity, and the burden to the health system associated with therapy. In this clinical scenario, strategies such as active surveillance have emerged that propose to manage low-risk BC with follow-up; however, due to the low evidence available, this is a strategy that is underutilized by clinicians. On the other hand, in the era of biomarkers, it is increasingly known how to use them as a tool in BC. Therefore, the aim of this review is to provide to clinical practitioners the evidence available to date on AS and the potential role of biomarkers in this therapeutic strategy in patients with low-grade/risk NMIBC. This is the first review linking use of biomarkers and active surveillance, including 29 articles.

## 1. Introduction

Bladder cancer (BC) is the tenth most common cause of cancer worldwide and is the thirteenth leading cause of cancer mortality. About 600,000 patients were diagnosed in 2020 with bladder cancer worldwide [1]. Non-muscle invasive bladder cancer (NMIBC) accounts for around 75% of new diagnoses [2]. Despite NMIBC progressing to muscle invasive or metastatic disease in approximately 15% of patients, NMIBC has a specific mortality rate less than 1% [2]. However, clinical evidence suggests that in patients with small (≤5 mm) and low-grade tumors, this progression risk is much lower [3].

Cystoscopy is the gold standard technique used to detect the presence of bladder cancer. If detected, a transurethral resection of the bladder (TURBT) is performed to remove the tumor and to determine its stage and grade. TURBT is the standard procedure for the diagnosis of bladder cancer and also a crucial therapeutic procedure for patients with NMIBC [4]. Although TURBT has been a technique of choice for decades with relatively low morbidity, it is far from perfect due to the 30% risk of understaging, and other complications. Approximately 4–6% of the cases show urinary tract infections and significant hematuria, but severe complications such as transurethral resection syndrome, active hematuria requiring endoscopic hemostasis, postoperative bleeding requiring blood transfusion, and wall perforation also occur [5,6]. Oncologic outcomes can also be compromised due to TURBT complications such as thermal damage at the lesion margin, absence of the detrusor muscle, tumor seeding, incomplete resection, or inaccurate pathologic assessment [5]. Additionally, complications after TURBT procedures and repeated cystoscopies over a long follow-up period impose a high and uncomfortable burden on patients, making BC one of the costliest malignancies in both economic and quality of life aspects [7]. It has been observed that the cumulative costs of 5 years of care per patient is $52,125 for low-risk cases, and $366,143 for high-risk NMIBC [8]. Reducing follow-up procedures such as cystoscopies and TURBT in low-grade/risk non-muscle invasive bladder cancer (NMIBC) patients is imperative to reduce the burden for patients, increase compliance, and reduce procedure complications. Additionally, over-reliance on follow-up procedures may strain healthcare resources and contribute to escalating public healthcare costs. Implementing adapted strategies that tailor surveillance intensity based on individual patient characteristics and tumor biology can optimize the balance between effective monitoring and minimizing patient burden and healthcare costs.

Low-risk NMIBC represents one of the costliest neoplasms to follow-up and treat due to its high recurrence rate [9]. Furthermore, some neoplasms are considered indolent, as they are stagnant or have a slower growth rate. It is in these cases when Active Surveillance (AS) takes a key role as a treatment strategy, as this approach ensures that resources are allocated efficiently while maintaining sufficiently high-quality care for patients [2]. In 2003, Soloway et al. suggested AS for selected recurrent LG NMIBC as a safe alternative to TURBT treatment and, despite the concept of AS still being fairly new in the NMIBC field, this strategy has been widely adopted successfully [10], making the strategy useful for low-risk BC and some intermediate-risk disease. However, there is still a need for consensus protocols to identify the most suitable patients for AS and predict both the likelihood of AS failure and disease progression [3].

Active surveillance has been established as a valid therapeutic choice that monitors BC cases with low progression risk which allows the avoidance or delay of the need to perform invasive treatments. This approach enables a reduction of the costs and morbidity associated with frequent TURBT. During the active surveillance period, the TURBT procedures are only performed after the exit from the AS protocol, for cases where disease progression is observed. However, patient selection and appropriate follow-up frequency are crucial to ensure the safety and effectiveness of this approach [11].

There is still no agreement on the appropriate active surveillance protocol and the optimal time to reduce the frequency of cystoscopy procedures. However, the majority of studies still suggest intensive follow-up (i.e., cystoscopy every 3 months) during the first year of AS, the period when most exits from AS occur. In some cases, this cystoscopy frequency is extended to a second year and then reduced to every 6 months [11,12]. One of the main advantages of AS lies in its potential to reduce the need for surgeries. When a LG NMIBC recurrent tumor is detected and excised, there is a significant likelihood of subsequent recurrences, which leads to more surgeries. By opting for AS upon the initial detection of recurrence, the need for a second surgery could be prevented.

Clinical and pathological inclusion or exclusion criteria are heterogeneously defined according to tumor volume, number of tumors, carcinoma in situ (CIS), or high-grade NMIBC (HG). In addition, absence of hematuria, negative urinary cytology, tumor volume <10 mm, and lesion number <5 are also used as cutoff points [13]. Among available studies of AS, the majority include pTa tumors, with only one study excluding pT1 tumors. Recently, Hurle et al. have proposed two modifications of the AS inclusion criteria during the pandemic period; increased tumor size and number of lesions up to a maximum of seven [9,14].

On the other hand, biomarkers have been used in bladder cancer for the diagnosis and prediction of recurrence and progression [15]. However, although the evidence is limited, some of the biomarkers already studied for diagnosis and recurrence, such as proteins, genes, and RNAs, could have a use in active surveillance [16]. These biomarkers have the potential to improve the sensitivity (SN) and specificity (SP) of current diagnostic methods and therefore reduce the need for or frequency of invasive procedures, such as cystoscopy or TURB, and monitor the inclusion and termination criteria for active surveillance [17]. In the era of precision medicine, future efforts should be focused on the validation of their sensitivity, specificity, predictive value, and their utility in follow-up and monitoring in AS and everyday clinical practice.

## 2. Objectives

The aim of this review is to provide to clinicals the evidence available to date on AS and the potential role of biomarkers in this therapeutic strategy in patients with low-grade/risk NMIBC.

## 3. Acquisition of Evidence/Methodology

The literature search was conducted using PubMed, Google Scholar, EMBASE, Wiley Library, and ClinicalTrials.gov. The reporting of this systematic review was guided by the standards of the Preferred Reporting Items for Systematic Review and Meta-Analysis (PRISMA) Statement [18]. The protocol was registered with the international prospective register of systematic review (PROSPERO), in accordance with PRISMA guidelines (PROSPERO Registration ID: CRD42024525890). The search was performed by two independent reviewers between November 2023 and February 2024 using the following keywords: Bladder cancer, Active surveillance, surveillance, monitoring, progression, NMIBC, biomarker.

We included original articles, studies with prospective and retrospective design, systematic reviews, and clinical trials from 2003 to 2023 involving AS or monitoring progression in NMIBC. Only articles in English were included; however, the search was not limited by language. We excluded letters, editorial, case reports (or with <10 subjects or samples), prevalence studies, protocols, narrative reviews or qualitative studies, meeting-driven poster/oral communications, articles with non-human models or in vitro studies, articles with insufficient description abstracts, and non-peer reviewed articles excepting clinical guidelines. In addition, articles that were duplicated on different platforms or with full texts that were unable to be accessed were also excluded.

Finally, publications that showed unsuitable participant criteria, unsuitable disease or treatment (only MIBC or CIS, multiple concurrent diseases or treatments), unsuitable technical outcomes (no sensitivity, specificity or AUC report), and/or unsuitable clinical outcomes (no report of prognosis, progression in stage, grade or muscle invasiveness) were excluded.

**Figure 1:**
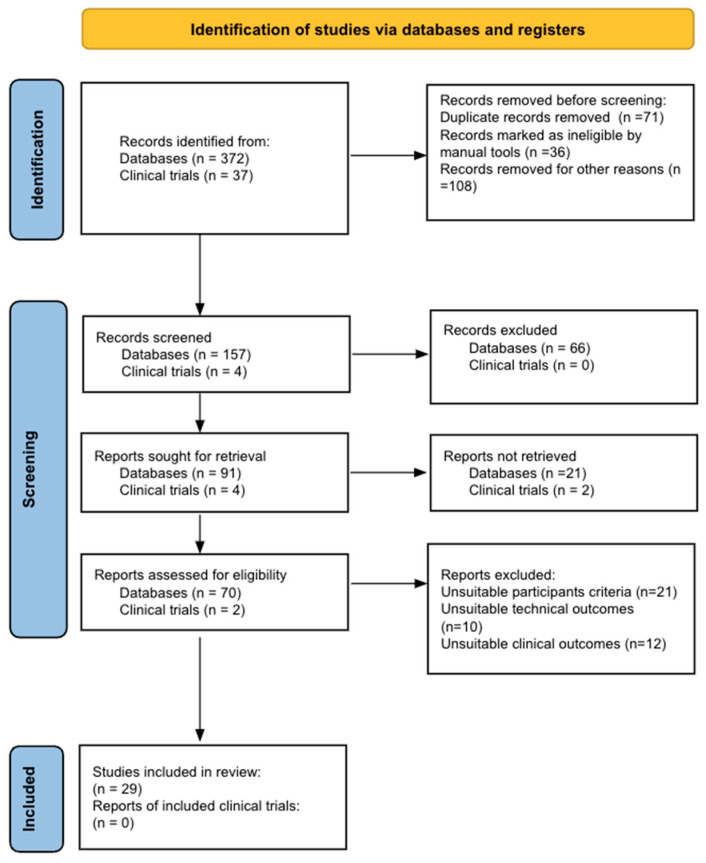
The flowchart on the stages of inclusion of studies in our systematic review according to the PRISMA guidelines.

After a first phase of evidence screening based on the evaluation of titles and abstracts, 73 articles were selected for independent evaluation of their full text by each of the reviewers. When there were disagreements on inclusion, these were resolved by a third reviewer. In addition, the reference lists of each of these papers were reviewed to identify additional studies. According to the inclusion and exclusion criteria, 29 articles were included. The results of the studies and the synthesis of each one are described in Tables and in the main text (narrative). This systematic review process is described in the PRISMA flow chart (Figure 1).

## 4. Synthesis of Evidence

The management of NMIBC aligns across various association guidelines, with the American Urological Association (AUA) and the European Urological Association (EUA) playing pivotal roles in guiding clinicians globally in their decision-making processes. These guidelines offer evidence-based recommendations regarding risk stratification, diagnostic procedures, treatment modalities, and surveillance strategies. According to the guidelines, for tumor recurrence both recommend complete resection of urothelial lesions if this is technically possible [19,20]. There are no specific recommendations associated with management of BC without lesion resection. Since it is not clear in these guidelines whether it is really possible to follow-up for the recurrence of lesions, some authors suggest that active surveillance could be safely performed in suitable patients.

According to the EUA guidelines, TURBT must be performed for all suspicious lesions in the urothelium if this is technically possible; if not, they strongly recommend active surveillance with cystoscopy in patients with low-risk tumors. In addition, these guidelines mention that biomarker-based medical devices, such as the XPERT BC^®^ MONITOR, EpiCheckTM, ADX BladderTM 0, CX BLADDER 0, and FDFGR3+TERT, have a promising role in follow-up after the resection of patients with NBCIM, but they mainly focus on those with high-grade tumors due to the low sensitivity rates (40–65%) reported for detecting recurrences in LG tumors [20,21,22].

The BIAS project [2], in order to avoid cystoscopy in patients with bladder cancer recurrences, included patients with 1–5 tumors sized less than 1 cm, with no hematuria, and with no history of HG tumors or CIS tumors or positive urinary cytology, and conducted active surveillance with a urine XPERT biomarker. Their results showed that this biomarker only missed 5.8% of recurrence tumors in patients without failing to meet AS criteria. These criteria were the growth of any lesion, the positiveness of any urinary cytology, the presence of hematuria, or the preferences of the patient. This study is mentioned in the EUA guidelines.

Regarding the AUA guidelines, in the same direction as the EUA guidelines, they treat every recurrence with resection, and emphasize that the main usefulness of biomarkers could be in the detection of recurrence in patients with high-grade tumors (AUA guideline) [19].

### 4.1. Active Surveillance

Active surveillance (AS) is a therapeutic alternative that can be proposed for patients with recurrence after an initial diagnosis of low-grade NMIBC: patients with low-grade pTa, less than 5 tumors, tumors of size ≤ 15 mm, and negative urine cytology, and who are asymptomatic and willing to accept closer surveillance, are suitable [23]. However, according to a review conducted by the EAU Young Academic Urology group, the level of evidence supporting AS is limited due to the available studies having heterogeneous inclusion criteria and follow-up procedures [24]. According to the French AFU Cancer Committee Guidelines, AS includes urine cytology and cystoscopy every three months in the first year and every six months thereafter. These AS protocols allow clinicians to avoid TURBT without compromising the oncological prognosis of monitored patients and also to include patients whose comorbidity does not allow surgical management or instillations [23]. However, this is the only clinical guideline that includes a detailed AS protocol as a management option for NMIBC, since other guidelines only briefly mention it or do not mention it [20].

Soloway et al. [10] published the first report of patients with NMIBC for whom their primary treatment strategy was observation. He included 32 patients. These patients had a history of Ta transitional cell carcinoma (TCC); tumor lesions were between 0.5–1 cm, recurrent, papillary, and low-grade. Only 3 (6.7%) of 45 observed tumors experienced progression from a non-invasive tumor (Ta G1 to 2) to a high-grade Ta or T1 tumor. None of the patients progressed to T2. Since then, studies have been carried out on active surveillance, but the inclusion and discontinuation criteria continue to be heterogeneous. We present the available evidence on active surveillance, which is summarized in Table 1.

Gofrit et al. (2005) [25] performed a retrospective study in 28 patients. The inclusion criteria were previous resection of low-grade Ta tumors (G1–2), no history of previous high-grade (G3) tumors, small papillary tumor (<10 mm), negative urinary cytology, and patient willingness to participate in AS. Of these, 18 patients had prior chemotherapy or immunotherapy. Cystoscopy and cytology were performed every 3 months for 2 years and then every 6 months. In total, 38 periods of AS were conducted in 28 patients. The mean duration of the period was 13.5 months, and 30 periods were concluded with tumor resection. The main reasons for surveillance discontinuation were the appearance of additional tumors (19 patients) and excessive tumor growth >10 mm (9 patients). The presence of hematuria indicated tumor removal in only one patient. All resected tumor masses were stage Ta (23 G1, 7 G2). Based on the previous results, it appears that small recurrent low-grade Ta papillary tumors pose a low risk for tumor progression, making active surveillance a safe practice.

Later, Pruthi et al. (2008) [26] retrospectively evaluated 173 patients with NMIBC. This study included patients with non-muscle invasive LG or HG papillary urothelial carcinoma, stage Ta or T1, or urothelial CIS. Exclusion criteria were urothelial papiloma, papillary urothelial neoplasm of low malignancy potential, or other atypical lesions. Among the participants, 22 cases (12.7%) underwent AS for bladder tumors in the prior 12 months. After TURBT, cystoscopy was performed every 3 months for the first 2 years, then every 6 months until 5 years, and then annually thereafter. All participants had a history of recurrent low-risk bladder tumors0, and were followed for a median of 25 months. During the follow-up of these 22 patients, 8 tumors did not grow, 9 had minimal growth, and 5 had moderate growth. Moreover, 77% had experienced multiple recurrences before conservative treatment, and 23% had a single tumor recurrence. Throughout an accumulated follow-up of 550 months, there were 32 recurrences, resulting in an overall recurrence rate of 0.70 recurrences per 12 months. When the observation period ended, 8 (36%) of the patients had a complete absence of tumors, attributed to regression or fulguration (six with Ta, and two with TIS), and 2 (9.1%) had disease progression (Ta to T1 and TIS to Ta), while 11 had persistent disease and stage. Additionally, smoking status was taken into consideration, where in smoking patients, the mean recurrence per patient was 2.6, compared to the mean recurrence of 0.8 per patient for non-smokers. The authors concluded that conservative treatment of recurrent bladder tumors is an appropriate option in patients with low-grade Ta tumors. Additionally, this strategy could avoid potential risks and morbidity associated with TURBT.

Hernandez et al. (2009) [27] conducted a prospective study of 273 patients with NMIBC. A total of 64 patients were included in the study, and there were a total of 70 observation events, as some patients were included in the observation more than once during the follow-up. The patients included in this study were those who had papillary tumors, negative urinary cytology, prior tumors at stage pTa, pT1, grade 1–2, less than 1 cm in size, and fewer than 5 tumors. Patients presenting with carcinoma in situ (CIS) or G3 tumors were not included. All patients included in the observation group underwent close monitoring with cytology and flexible cystoscopy every 3–4 months. A retrospective analysis of a control group of patients with clinical characteristics similar to those of patients under active surveillance was also performed, but they underwent immediate transurethral resection after the diagnosis of recurrence. Data from 64 patients (70 observation events, defined as time of patient entry to AS until they underwent TURBT) were analyzed. Patients were followed for a median of 38.6 months, and the median observation time was 10.3 months. Participants included in this article were pTa (77.1%), pT1a (22.9%), G1 (67.1%), and G2 (23%). After a median follow-up of 10.3 months, 93% did not progress in stage and 83% did not progress in grade. None progressed to T2 (0%). In contrast, in the control group, where immediate transurethral resection was performed at diagnosis, 2 patients progressed to stage T2. In the study group, patients who experienced an increase in the number and/or size of lesions (less than 1 cm, and/or <5 tumors), hematuria, or positive urinary cytology for malignancy discontinued the AS period. The study concluded that patients with small tumors <1 cm and non-muscle invasive tumors can safely be offered the active surveillance protocol, reducing the number of future interventions.

In 2016, Hurle et al. [28] conducted a retrospective study on 293 patients with low-grade (pTa–pT1a) NMBC (pTa–pT1a), of which only 55 met the inclusion criteria. Their inclusion criteria were negative urinary cytology, <5 lesions with a diameter <10 mm, and absence of CIS or persistent hematuria. No patients with a history of a high-grade carcinoma (Grade 3), CIS, or positive cytology findings were included in this study. In total, the study included 55 patients with a total of 70 AS events (some patients entered the observation program multiple times throughout their follow-up period). The mean age of the patients was 69.8 years. The median follow-up was 53 months. The median time patients remained on AS was 12.5 months. Disease progression occurred in 28 patients (51%), from low to high grade, but no patients progressed to T2 stage. Overall, 15 patients (27.3%) showed an increase in the number and/or size of tumors, nine (16.4%) had hematuria, and four (7.3%) had positive cytology. Only five (9%) patients in the entire series progressed to high-grade (Grade 3) tumors or presented associated CIS. The overall adherence to the follow-up program was 95%. The authors concluded that the AS protocol for NMIBC could be a reasonable alternative in patients with recurrent low-grade pTa/pT1a small papillary bladder tumors; on the other hand, AS reduces the number of interventions that patients will need during their lifetime and avoids therapy-induced complications.

Hernandez et al. (2016) [29] studied AS in a retrospective cohort of 186 patients from 1999 to 2014. The inclusion criteria were the presence of recurrent papillary tumors, stages pTa–pT1, with previous grades G1–G2, less than 1 cm, and fewer than 5 tumor locations. Exclusion criteria were HG carcinoma, CIS, and urinary cytology positive. Patients were followed up with urinary cytology and flexible cystoscopy every 3 months for one year and then every 6 months. Previously, 80 patients received adjuvant therapy with mitomycin C and Bacillus Calmette-Guérin (56 and 24 patients, respectively). The tumor characteristics of the patients before enrollment were 131 TaG1 (51.9%), 54 TaG2 (21.4%), 25 T1G1 (9.9%), and 42 T1G2 (16.7%). Mean time from last TURBT to enrollment in the surveillance program once recurrence was detected was 11.8 months, and mean time from the first TURBT was 24.3 months. Of all periods, active treatment was performed in 203 (80.6%) cases, and the median of treatment-free survival after recurrence diagnosis was 13.4 months. After observation, 171 surveillance periods (86.4%) showed no progression in stage, and 157 (79.3%) showed no progression in grade. Among these patients, 9 (3.6%) progressed to G3, 6 (2.4%) progressed to CIS, and 4 progressed to T2; these patients previously had T1G2 tumors smaller than 3 mm, and underwent radical cystectomy when progression was detected, with only three of them showing an increase in the number of tumors and one a positive cytology. In their analysis, the authors observed that associated factors with a higher risk of grade progression were multiple lesions, previous stage and grade, age, and time since the initial TURBT; however, they were not related to tumor stage progression.

The most recent work that shows the role of AS was Contieri et al. in 2022 [3] who conducted a retrospective study in 214 patients. The inclusion criteria were patients with ≤5 suspicious lesions at recurrence, maximum diameter of the lesion less than 1 cm, absence of gross hematuria, and negative urinary cytology. Exclusion criteria are patients with a history of a HG carcinoma (Grade 3), CIS, or positive cytology findings. Failure criteria were the development of any exclusion criteria during follow-up, or voluntary withdrawal by patient decision. For patient follow-up, the authors performed a flexible cystoscopy and urinary cytology every 3 months for one year, and then 6 months annually. The median follow-up and time on AS were 38.8 and 13 months, respectively. In 90 cases (35.8%), patients received intravesical treatment before enrollment in AS, with mitomycin C in 55 cases and Bacillus Calmette-Guérin in 25. Out of 251 AS events, 130 cases (51.8%) experienced AS failure and underwent TURBT, either due to tumor size increase (n = 51, 39.2%), number of lesions (n = 34, 26.1%), increase in both number and size of lesions (n = 29, 22.3%), positive cytology (n = 11, 8.5%), or gross hematuria (n = 3, 2.3%). In only two cases (1.5%), patients requested voluntary withdrawal. In the main results of this study, the probability of active treatment absence was 59.7%, 54.5%, 46.3%, and 40.4% at 12, 18, 24, and 36 months, respectively. A total of 95 patients (37.8%) remained on AS for more than 18 months, with a median AS duration of 33 months. During a follow-up of 44.6 months, 23 therapy failures were observed. There were no significant differences in the population based on whether they remained on AS> or <18 months; however, the proportion of patients who underwent two or more TURBT procedures before AS enrollment was significantly lower in the >18-month AS group (19.8% vs. 37.8%; *p* = 0.004), as well as the proportion of patients with two or more lesions at AS enrollment (28.4% vs. 45.5%; *p* = 0.007).

In addition, to address the complications derived from frequent cystoscopies in active surveillance and follow-up, some studies have suggested non-invasive methods [30,31]. Regarding active surveillance, Konstantinos Stamatiou et al. (2011) [31] performed a study with 33 patients with a recent history of recurrent superficial BC and subsequent TURBT being under active surveillance by both transabdominal ultrasound and cystoscopy methods. Ultrasound is a non-invasive and cost-effective technique that can be safely performed on all individuals with no restrictions. The study reported that the sensitivity of ultrasonographic techniques for detecting recurrence was 78.5%, the specificity 100%, and the negative predictive value 86.3%. However, lesions smaller than 0.5 cm and lesions located in the dome or bladder neck showed to be more difficult to visualize sonographically [31]. Ultrasound may be useful in follow-up of patients under AS protocol; however, it still cannot replace cystoscopy due to its reduced sensitivity compared to cystoscopy.

**Table 1:** The following table shows the active surveillance studies carried out to date, taking into consideration the inclusion criteria, exit criteria and average follow-up and stages included.

Authors	Stages	N	Study	Inclusion Criteria	Exit Criteria	Follow-Up Period *	Conclusions
Soloway et al., 2003 [10]	Ta or T1 G1–G2	T = 32 Not control group	Retrospective	Small día-meter: 0.5–1.0 cm Papillary tumor Low Grade	Not specify	10.09 months	Small, recurrent, low-grade appearing bladder tumors are slow growing and pose minimal risk.
Gofrit et al., 2005 [25]	Ta tumors (G1–G2)	T = 28 Not control group	Retrospective	No history of previous high grade (G3) tumors, small papillary tumor (<10 mm) negative urinary citology	Additional tumors Excessive tumor growth >10 mm		Small recurrent low-grade Ta papillary tumors possess a low-risk tumor progress, making active surveillance a safe practice.
Pruthi et al., 2008 [26]	Superficial bladder cancer	T = 173 22 patients were included Not control group	Retrospective	Noninvasive low- or high-grade papillary urothelial carcinoma (Ta, T1) or urothelial carcinoma in situ	Urothelial papilloma Papillary urothelial neoplasm low malignancy potential Atypical lesion	25 months	Conservative treatment of recurrent bladder tumors is an appropriate option in patients with low-grade Ta tumors.
Hernandez et al., 2009 [27]	Pta, pT1	T = 273 64 patients were included 203 were control group	Prospective	Papillary tumors Negative cytological findings Less than 1 cm in size Fewer than 5 tumors	Increase in the number and/or size of lesiones (less than 1 cm, and/or <5 tumors) Gross hematuria Positive urinary cytology	38.6 months	Small tumors <1 cm and non-muscle invasive tumors can safely be offered the active surveillance protocol.
Hernandez et al., 2016 [29]	pTa–pT1 G1–G2	T = 186 Not control group	Retrospective	Recurrent papillary tumors Less than 1 cm in size Fewer than 5 tumor locations	Increase in the number or size of the lesions Symptoms (mainly hematuria) Positive urine cytology	53 months	Associated factors with a higher risk of grade progression were multiple lesions, previous stage and grade, age, and time since the initial TURBT, however, they were not related to tumor stage progression.
Hurle et al., 2016 [28]	pTa–pT1a	T = 293 55 patients were included not control group	Retrospective	Urinary citology (−) <5 lesions A diameter <10 mm Absence of CIS ª or persistent hematuria	Positive cytology CIS up-grade	53 months	AS protocol for NMIBC could be a reasonable alternative in patients with recurrent low-grade pTa/pT1a small papillary bladder tumor.
Contieri et al., 2022 [3]	pTa (grade 1–2) and pT1a (grade 2)	T = 214 156 patients ≤18 months of AS ° 95 patients >18 months of AS °	Retrospective	≤5 suspicious lesions Diameter ≤ 1 cm Absence of gross hematuria Negative urinary cytology	Tumor size increase > 1 cm Number of lesions ≥ 5 Increase in both number and size of lesions Positive cytology Gross hematuria	36.8 months	Well-selected patients with NMIBC can safely remain on AS for a long period of time. Multiple TURs and multiple lesions at AS enrollment are associated with a higher risk of AS failure.

* Relative time lapse associated with AS exit and/or progression; ° Active Surveillance; ª Carcinoma in situ. AS protocol for NMIBC could be a reasonable alternative in patients with recurrent low-grade pTa/pT1a small papillary bladder tumors.

### 4.2. Biomarkers

Biomarkers may play a crucial role in detecting bladder cancer recurrence and progression. These molecular indicators provide valuable insights into the biological characteristics of cancer cells, enabling clinicians to identify and assess the severity of the disease [32]. Several biomarkers have been identified through genomic analysis, proteomic analysis, and gene expression profiling. Urine-based liquid biopsy has emerged as a non-invasive and effective tool for early screening and diagnosis of bladder cancer [33]. Biomarkers used for bladder cancer include tumor DNAs, proteins, microbiome, tumor RNAs, long non-coding RNAs, transfer RNA-derived fragments, messenger RNAs, microRNAs, circular RNAs, exosomes, and extrachromosomal circular DNA [34]. Recent investigations into the molecular landscape of bladder cancer have revealed frequent genetic alterations and molecular subtypes with therapeutic implications. Several biomarkers have been highly developed and tested to be used for early diagnosis or predict response to treatment, but only a few have been associated with recurrence or active surveillance [35]. Integrating biomarker analysis into bladder cancer management and active surveillance by helping identify patients at risk of recurrence or progression will improve quality of life and reduce frequency of invasive procedures, disease-associated costs, and healthcare burden by guiding treatment decisions by selecting personalized strategies [36].

How could biomarkers be used in AS? Today, most studies have been focused on urine biomarkers, and evidence suggests that these could contribute to main aspects of AS, such as inclusion and exclusion criteria of the protocol, and together with cystoscopy, improve its ability to detect recurrence and progression or failure to AS criteria. This is summarized in Table 2.

Regarding urine biomarkers that could serve to refine inclusion and exclusion criteria in AS, we identified different articles that show this potential role:

DNA methylation: Sheng-Fang Su et al. [37], in 2014, analyzed the presence of the DNA methylation of 6 biomarkers. For this purpose, they analyzed 368 urinary sediment samples from 90 patients diagnosed with non-muscle invasive bladder cancer at any stage (Tis, Ta, T1; grade low–high). These 6 biomarkers included hypermethylation of HOXA9, SOX1, NPY, IRAK3, and ZO2, and hypomethylation of L1-MET. In all cases of hypermethylated genes, a statistically significant increase in expression was demonstrated when compared to controls; for the case of the L1-MET gene, there was decreased expression, also with a statistically significant difference from controls. In order to create a kit with the best predictive results for recurrence, they constructed a logistic regression model that determined that combination of hypermethylated SOX1 and IRAK3 genes plus the hypomethylated L1-MET gene would have the best predictive results. In an internal validation test, a combination of these three markers would have a sensitivity of 80% and a specificity of 97% for detection of recurrences. On the other hand, they mentioned that 80% (16/20) of samples positive for the presence of methylated DNA finally recurred, while 74% (52/70) of the samples negative for presence of methylated DNA did not recur. Comparing these results, they concluded that the predictive potential of this test would be around 80%, versus 35% for cytology. For AS purposes, this biomarker could be used to assess when to use additional cystoscopy.

FGFR3 mutation and methylation: JP Roperch et al. [38] in 2016 performed a 2-year follow-up study on 455 urine samples from 158 patients with a primary NMIBC tumor (pTa, pT1, CIS, low or high grade) treated by TURBT (81% males). DNA was isolated from urine samples for detecting both FGFR3 mutation (S249C, Y375C, R248C, and G372C) and hypermethylated genes (HS3ST2, SEPTIN9, and SLIT2) by allele- and methylation-specific PCR, respectively. Out of the 425 follow-up urine samples, 353 were recurrence-free; by this combined biomarker, the detection of recurrence was 96.4% pTa, 100% pT1, 100% CIS, 50.0% other tumor stages, and 93.6%/96.0% low-/high-grade tumors. A total of 68 correctly predicted recurrences were distributed in the low/intermediate/high-risk group as 93.3%/92.9%/96.6%, which indicates a higher propensity of the test to detect high-risk patients. Finally, the authors obtained a sensitivity of 94.5%, a specificity of 75.9%, and a NPV of 98.5%, with an AUC of 0.82 on the on the whole surveillance set. This biomarker serves as a pivotal tool in identifying recurrency of bladder cancer, presenting a promising avenue for enhancing active surveillance protocols by potentially suggesting the need of cystoscopy, refining cystoscopy results and re-evaluating patients’ inclusion criteria

mRNA sequences: Elsawy et al. (2021) [39] assessed the clinical performance of the Xpert Monitor test for recurrence detection during surveillance of NMIBC patients. The study included patients who had NMIBC and were scheduled for cystoscopy and excluded patients who had a history of CIS, recent excision procedure (TURBT), or intravesical BCG/chemotherapy treatment within 4 weeks. Prior to cystoscopy, a urine sample was collected and cytology was evaluated. In positive or suspicious cystoscopies, biopsy or TURBT was performed after office cystoscopy to confirm or exclude recurrence. In these patients, the GeneXpert system was used to detect ANXA10, UPK1B, CRH, and IGF2 mRNA in urine samples by RT-PCR. Regarding Xpert Monitor, the overall sensitivity, specificity, and negative predictive values were correlated to cytology, being 73.7%, 79.6% and 96.3%, respectively. Additionally, cystoscopy-negative patients were followed up by regular cystoscopy according to the standard-of-care protocol for a median period of 9 months (range: 5–19 months) and recurrence was observed only in 15 (9.3%) patients. Interestingly, the Kaplan–Meier and Cox curves between tumor grade and Xpert Monitor results were significantly associated with early tumor recurrence. For AS purposes, this biomarker could be used to assess the appropriate time to perform additional cystoscopies.

Regarding urine biomarkers related to progression that could improve failure or exit criteria in AS, we identified different articles that evidence this potential role:

ucfDNA: Birkenkamp-Demtröder K et al. [40], in 2016, performed a long-term retrospective pilot study with 377 tumor, urine, and blood samples from 12 patients diagnosed with non-muscle invasive bladder cancer, with aim of studying the presence of urinary cell-free DNA (ucfDNA) as a follow-up method for progression of non-muscle invasive disease. These were separated into two equal groups; one group of patients with progression to invasive or metastatic disease (PRO) and another group with recurrence of non-muscle invasive tumor (REC). The main objective of this work was to look for the presence of cfDNA in urine, to be used as a follow-up method for progression of non-muscle invasive disease. With a follow-up of 20 years, authors demonstrated that tumor-specific ucfDNA was detected in 96.5% of the PRO group, and only in 50% of the REC group. Elevated levels of tumor-specific ucfDNA were observed in all patients in the PRO group. Of these, in 83% of cases detection was made several months before clinical progression to invasive muscle disease (range of 33–223 months), even when there was very low or undetectable cfDNA in plasma; this suggests that ucfDNA may be more sensitive than plasma in the detection of bladder cancer. Although the authors conclude that ucfDNA detection would not replace the use of cystoscopy, it would be a useful method for predicting tumor behavior in terms of aggressiveness, progression, and invasion, and would therefore be useful as a method for early detection of progression and metastasis. This biomarker was detected early in patients in the PRO group, so it could be useful for contraindication or as a failure criterion for AS.

ucfDNA: Xu et al. [41], in 2019, analyzed urinary cell-free DNA (ucfDNA) as a potential biomarker for prognosis in 103 patients with NMIBC and identified a model combining protein ratios of IQGAP3/BMP4 and IQGAP3/FAM107A associated with disease grade in NMIBC. Overexpression of IQGAP3/BMP4 was associated with higher grades and stages (*p* = 0.015 and *p* = 0.005, respectively) as well as recurrence and progression (*p* = 0.002 and *p* = 0.001, respectively), whereas IQGAP3/FAM107A was associated with greater tumor size and progression (*p* = 0.019 and *p* = 0.001, respectively). Notably, results showed that tumor grade (*p* = 0.010) and IQGAP3/BMP4 (*p* = 0.004) were independent risk factors for progression. Regarding prognosis, there was a significant difference between higher levels of IQGAP3/BMP4 with worse RFS (*p* = 0.001). PFS was associated with increased expression levels of both IQGAP3/BMP4 and IQGAP3/FAM107A (*p* < 0.001 and *p* = 0.006, respectively). This biomarker was associated with disease grade; thus, it could be useful for detecting failure criteria for AS

CircRNAs: Song et al. (2020) [42] analyzed a novel circRNA, hsa_circ_0137439, in 116 urine samples from 10 bladder cancer patients and 30 urine samples from 10 non-cancer patients. Their results indicate that a high expression of hsa_circ_0137439 was related to clinical progression of bladder cancer, being higher in advanced tumor stages (*p* < 0.001), higher grade (*p* < 0.001) and higher lymph node status (*p* = 0.035), and MIBC (*p* < 0.001). Regarding usefulness in AS, the increase of these biomarkers could suggest the need to perform surveillance cystoscopy, to re-evaluate AS criteria, and to suspect tumor upgrade or upstage, indicating the need for TURBT. The identification of progression is fundamental to making AS a safe option.

NDRG2 gene: Miao Zhang et al. (2017) [43] assessed the potential clinical utility of N-Myc downstream-regulated gene 2 (NDRG2), which has been associated with cell differentiation and proliferation, in discriminating bladder cancer in a total of 124 patients who had not received any chemotherapy or radiotherapy before sampling (71 with tumor stage T1–T2 and 45 T3–T4), and 97 healthy controls matched by age and sex. A voided midstream urine sample was obtained from all subjects before cystoscopy and also from healthy controls, and both relative mRNA expression and protein levels of NDRG2 were calculated by qRT-PCR and Western blot, respectively. NDRG2 expression was significantly lower in patients with bladder cancer compared to healthy controls. The ROC curves indicated that NDRG2 had a high diagnostic value (with a cut-off point of 4.840), showing an AUC of 0.888, a sensitivity of 85.5%, and a specificity of 81.4%. Specifically, a significant association was found between low NDRG2 expression and tumor grade, as well as disease stage. To consider NDGR2 expression in active surveillance could augment the accuracy from urine cytology or cystoscopy, and therefore provide improved inclusion criteria for patients.

lncRNA: A prospective study of Lutao Du et al. (2018) [44] recruited a total of 240 bladder cancer patients with stages Ta–T1 (79 patients) and T2–4 (41 patients) and 240 controls to assess to role of long non-coding RNAs (lncRNAs), 200 nucleotides with low protein-coding potential, in bladder cancer. lncRNAs have been reported to be involved in both tumorigenesis and progress. Urine samples were obtained before patients underwent TURBT or radical cystectomy. Then, lncRNAs were isolated and urine cytology was also performed to assess the diagnostic value of the biomarker, and patients were monitored every 3 months for the first 2 years, and then every 6 months until completion of 5 years. After the discovery and validation of two IncRNAs, uc004cox.4 and ENST00000414075 (GAS5), significant changes were observed between patients with BC and the control group; uc004cox.4 was up-regulated and GAS5 was down-regulated in BC patients, reaching AUC values even higher than urine cytology; 0.799 and 0.767, respectively. Remarkably, a higher urinary level of uc004cox.4 was significantly correlated with advanced tumor stage (*p* < 0.05), but no associations were found between the two lncRNAs and tumor grade and lymph node metastasis. In the NMIBC group, but not in the MIBC one, Kaplan–Meier and Cox analysis showed that higher expression level of uc004cox.4, but not GAS5, and tumor stage were associated with poorer recurrence-free survival (RFS). The usefulness of this biomarker in AS would suggest the need to perform surveillance cystoscopy and support exit criteria of patients.

miRNA: In the study of Mamdouh S. et al. (2022) [45], both urine and tissue samples from 111 bladder cancer patients without any type of therapy (male 89.2%, 39 low-grade and 72 high-grade urothelial carcinomas) and from 25 healthy age- and sex-matched controls (males 72%) were included. The miRNAs miR-200, miR-145, and miR-21 were isolated from urine pellets and quantified by qRT-PCR. The three miRNAs’ expression levels were significantly upregulated in the urine and tissue of non-muscle invasive bladder cancer patients compared to controls. In addition, both urinary miR-200 and miR-145 levels serve for tumor grade stratification. miR-200 has a cut-off value of 2.789 (AUC of 0.699), sensitivity of 41.7%, and specificity of 100.0%, whereas miR-145 has a cut-off value of 0.042 (AUC of 0.702), with 91.7% sensitivity, and specificity of 46.2%. In addition, urinary miR-145 expression was associated with clinicopathological outcomes such as positive lymph nodes, Squamous cell carcinoma, and patients with GIII. Regarding urinary miR-21, despite no significant association being observed with clinicopathological outcomes, its levels were able to discriminate by tumor grade with a cut-off value of 0.083 (AUC value of 0.647), 95.8% sensitivity, and specificity of 38.5%. For AS, the increase of these biomarkers could suggest the need to perform surveillance cystoscopy, as the suspicion of tumor upgrade is a crucial safety parameter in AS that could be considered as a failure criterion.

**Table 2:** This table summarizes the articles reviewed about biomarkers and their potential use in active surveillance.

Author	Biomarker	Sample	Molecule	Technique	N	Usefulness in Active Surveillance
A Schneider et al., 2000 [46]	iD9S162, IFNA, D16S310, D16S476, D4S243, FGA, ACTBP2, D9S171, D9S747, MJD52, D8S307, THO, D13S802, D17S695, D17S654, D20S48, TP53	Urine and Blood	Microsatellite DNA	RT-PCR	T = 209 103 BC, 80 other disease and 26 controls	Recurrence SN = 84%
Robert S Svatek et al., 2006 [47]	sFAS NMP22	Urine	RNA	ELISA	T = 229 122 BC and 107 controls	Progression Stage SN = 75%
Sheng-Fang Su et al., 2014 [37]	HOXA9, SOX1, NPY, IRAK3, and ZO2,f L1-MET.	Urine	DNA	Pyrosequencing	T = 90 56 BC patients without recurrence and 34 BC patients with recurrence	Recurrence SN = 80%
Birkenkamp-Demtröder et al., 2016 [40]	4–48 personalized genomic variants	Urine and plasma	ucfDNA	RT-PCR	T = 12 6 BC patients with progression and 6 BC patients with recurrence	Progression
Roperch J. et al., 2016 [38]	FGFR3 S249C	Urine	DNA	Methylation-specific RT-PCR	T = 263 158 BC and 105 controls	Recurrence SN = 97% SP = 84.8%
Ye-Hwan Kim et al., 2016 [48]	Topoisomerase-II alpha (TopoIIA)	Urine supernatant	cfDNA	RT-PCR	T = 198 83 BC, 54 patients with hematuria and 61 controls	Exclusion criteria SN = 73.8% SP = 68.3%
Fantony et al., 2017 [49]	NID2 TWIST1	Urine	DNA	Methylation-specific RT-PCR	T = 172 63 patients with hematuria and 109 NMIBC patients	Recurrence SN = 54%
Zhang et al., 2017 [43]	NDRG2	Urine	RNA	RT-PCR Western blot	T = 221 124 BC and 97 control subjects	Progression Grade Stage SN = 85.5% SP = 81.4%
Van der Heijden A. et al., 2018 [50]	CFTR, SALL3, and TWIST1	Urine	DNA	Pyrosequencing	T = 168 111 BC and 57 controls	Recurrence SN = 96% SP = 40% NPV = 92%
Allione A. et al., 2018 [51]	MMP23A MMP23B	Urine	Protein	ELISA Western blot	T = 101 44 BC and 57 controls	Recurrence
Lutao Du, et al., 2018 [44]	GAS5 uc004cox.4	Urine	IncRNA	qRT-PCR Microarray analysis	T = 480 240 BC and 240 controls	Progression Identify high-risk tumors SN = 84.5% SP = 78.2%
Thorsten H Ecke et al., 2018 [52]	Cytokeratin 8 Cytokeratin 18	Urine	Protein (soluble fragments)	ELISA	T = 530 182 NMIBC patients, 60 MIBC, 62 patients with non-evidence of disease and 226 controls	Progression Identify high-grade vs. low-grade tumors SN = 78.8% (Low grade) SN = 75% (High grade) SP = 93.8%
Hofbauer S. et al., 2018 [53]	6 miRs (let-7c, miR-135a, miR-135b, miR-148a, miR-204, miR-345)	Urine	RNA	RT-PCR	T = 245 133 BC and 112 controls	Recurrence SN = 94% SP = 51%
Yanjie Xu et al., 2019 [41]	IQGAP3 BMP4 FAM107A	Urine	uctDNA	RT-PCR	103 BC	Recurrence Progression stage and grade
Yujiro Hayashi et al., 2020 [54]	TERT C228T	Urine	ucfDNA	ddPCR RT-PCR	T = 76 40 pre-TURB patients and 36 surveillance group	Recurrence SN = 68.9% SP = 96.2%
Xu Chen et al., 2020 [55]	2 CpG markers (cg21472506 and cg11437784)	Urine	DNA	utMEMA	T = 175 109 BC and 66 controls	Recurrence SN = 90% SP = 83.1%
Song Z et al., 2020 [42]	hsa_circ_0137439	Urine	circRNA	Microarray analysis, RT-PCR	T = 146 116 BC, 30 controls	Progression, predict grade, stage and lymph node status
Zhenyu Ou et al., 2020 [56]	Urinary cell-free DNA (ucfDNA) TERT, FGFR3, PIK3CA and KRAS	Urine	cfDNA	Next-generation sequencing	T = 125 92 BC and 33 controls	Recurrence
Elsawy et al., 2021 [39]	ANXA10 UPK1B CRH IGF2	Urine	RNA	Xpert monitor	181 NMIBC	Recurrence SN = 73.7% SP = 79.6% NPV = 96.3%
Leihong Deng et al., 2022 [57]	*DMRTA2*	Urine	DNA	Methylation-specific RT-PCR	T = 127 44 BC, 83 controls	Recurrence SN = 82.9% SP = 92.5%
Anouk E. Hentschel, 2022 [58]	FAM19A4, GHSR, MAL, miR-129, miR-935, PHACTR3, PRDM14, SST and ZIC1	Urine	DNA	Methylation-specific RT-PCR	T = 26 14 BC and 12 benign hematuria patients	Recurrence SN = 80% SP = 93%
Samah Mamdouh et al., 2022 [45]	miR-200, miR-145 miR-21	Urine	RNA	RT-PCR	T = 136 11 BC and 25 controls	Recurrence Progression, discriminate high grade v/s low grade SN = 91.7% SP = 46.2%
TOTAL					4013	

circRNA, circular RNA; ddPCR, droplet digital polymerase chain reaction; ELISA, enzyme-linked immunosorbent assay; RT-PCR, real-time polymerase chain reaction; SN, sensitivity; SP, specificity; NPV, negative predictive value; BC, urothelial bladder carcinoma; ucfDNA, urinary cell-free DNA; uctDNA, urinary circulating tumour; utMEMA, a method detect urine tumour; T, total of patients. NA, not applicable.

## 5. Discussion

The first active surveillance (AS) study was conducted in 2003 by Soloway [10]. Now, almost 20 years later, the evidence is still scarce, with only seven studies assessing this management strategy. Therefore, it is barely approached in international guidelines [19,21,23,24]. Additionally, since disease prognosis deteriorates significantly when NMIBC progresses to MIBC or metastatic disease, clinicians usually choose a strategy based on the risk in the management of NMIBC patients [19,20,35]. However, it is known that only 15% of the NMIBC cases will progress to MIBC or metastatic disease, and the persistent surgical treatment for recurrence events leads to a heavy burden regarding QoL, complications, comorbidities, and economic costs [16,59]. A well-executed AS protocol could improve both patient and healthcare service burdens, decrease bladder cancer-associated costs, and reduce TURBT-driven morbidity without affecting the oncologic outcomes of the patients [9,10]. Nowadays, it is crucial to explore treatment options that allow physicians to be certain about their decision-making and patients to feel safe and monitored, avoiding both under- and over-treatment. The available evidence of AS protocols shows an upgrade rate of 9.1–51% and an upstage rate of 6.5–13.6% [3,10,14,25,26]. A single study observed 4/186 patients who progressed to T2 (2.15%) [27].

Recently, the clinical use of biomarkers in bladder cancer has been mainly described in diagnosis and prediction of treatment response [16,32,33]. In addition, a certain amount are also able to identify early recurrence and progression, a feature that could improve the accuracy of cytology, the identification of requirement for cystoscopy or TURBT, and the selection of patients who could actually benefit from surveillance by precising both the inclusion and exit criteria [16,60]. Moreover, most of the biomarkers that could be used in AS are focused on detecting recurrence and not progression [16,33,35,45]. However, this type of biomarker might also be useful for the identification of the most effective time to perform an additional cystoscopy. This approach will allow physicians to detect cases with higher risk of progression early, while also evaluating the extension of the period in-between cystoscopies during follow-up [17].

Several biomarkers were able to identify upgrade and/or upstage of tumors according to a cut-off value, which may be useful in AS to detect exclusion criteria, the need for cystoscopy, and monitoring the interruption of the protocol, among other uses [16,34,61]. Remarkably, tumor ucfDNA seems to be a promising biomarker in AS, as it was found in 96.5% of the cases with tumors that progressed, and in 83% of them it could be detected several months before invasion actually occurred [40]. Therefore, it is a biomarker that can facilitate discard or exit from AS at a certain range that suggests risk of progression [40]. Biomarkers are an innovative approach in oncology as a personalized strategy [32]. In AS, biomarkers could improve management protocols’ safety by refining the patients’ criteria for inclusion, exclusion, and exit, therefore reducing the chance of patients developing progression during AS. An AS strategy could reduce overtreated cases, thus decreasing the costs associated with comorbidities triggered by multiple TURBTs. Additionally, the use of biomarkers in AS could reduce undertreatment cases, increasing patient safety and decreasing disease-associated risks.

Despite this, the field is still under exploration, and new biomarker studies emerge constantly [32,34,62]. Research into new AS protocols including biomarkers should be encouraged, achieving a balance between management of patients with bladder cancer avoiding both overtreatment and undertreatment, and reducing the burden associated with healthcare and financial costs driven by this disease. At the moment, there are limited clinical trials including AS protocols. Among those, the clinical trial NCT02700724 entitled “Observation Versus Immediate Surgery of Low-Risk Bladder Cancer” was terminated early due to poor enrollment [63]. Furthermore, the clinical trial NCT02298998 entitled “Surveillance for Low and Low-Intermediate Risk Non-muscle Invasive Bladder Cancer: A Pilot Study”, although it is focused in surveillance and not active surveillance, compares associated costs and QoL regarding clinical procedures between high-frequency (EAU guideline) and low-frequency surveillance (AUA guidelines) protocols [64]. Published results show that both groups had similar patient-reported procedure-related discomfort and quality of life measures over time. However, as expected for high-frequency surveillance, patient out-of-pocket costs and healthcare systems costs were $383.80 more per patient annually in the high-frequency surveillance group compared to the low frequency group [65]. Interestingly, the clinical trial NCT05148728 entitled “Active Surveillance vs in Office Fulguration for Low Grade Bladder Cancer Tumors” will compare clinical outcomes as progression and complication rates, including an active surveillance group [66]. Results derived from these trials could support the effectiveness, benefits, and safety of AS.

The limitations of the review processes used are based on human work that could eventually be objectively standardized through the use of software. The main limitation of this review topic relies on the diversity among existing studies on active surveillance; variations in inclusion, exclusion, and failure criteria, together with different follow-up protocols, which impair the comparison between studies. On the other hand, we do not know the specificity of most of the biomarkers, because in the body of the articles, although sensitivity is studied, we do not have data on specificity. It is a subject that is still under study and it is very new in BC; therefore, there are a large number of potential biomarkers, so it is necessary to study which ones could have a greater clinical use, and the costs associated with the use of each biomarker.

## 6. Future Directions

Bladder cancer, particularly in low-grade non-muscle invasive cases, poses a significant problem due to its potential for recurrence and progression [67]. Morbidity and costs associated with bladder cancer underscore the need for effective surveillance strategies to monitor mainly disease progression, rather than recurrence [68]. However, despite numerous biomarkers being investigated, only a limited number have gained approval from the FDA for clinical use. The multitude of biomarkers released for diagnosis reflects the intense research interest in this field, yet their utility in surveillance remains ambiguous and without a specific recommendation [69]. Nonetheless, our study suggests that certain diagnosis biomarkers may have a crucial role in active surveillance for particular patients, thus potentially refining surveillance strategies and improving patient outcomes. If we succeed in establishing AS as the recommended strategy for low-risk, and some intermediate-risk, bladder cancer patients through a combination of clinical and biomarker criteria, we will be implementing the same approach that occurs nowadays for prostate cancer [70]. Further research, such as randomized clinical trials, is needed to validate these biomarkers’ clinical utility, paving the way for personalized and effective surveillance protocols in LG NMI bladder cancer patients.

## 7. Conclusions

Even though there are few studies and guidelines referring to active surveillance in patients with low-risk NMIBC, there is a need to make the follow-up more cost effective, especially in patients that are asymptomatic. The absence of inclusion or progression/intervention criteria to AS opens the possibility for the use of biomarkers, which can improve the role of AS, and diminishing the overtreatment, cost, and Qol burden for patients with bladder cancer.

## Data Availability

The original data presented in the study are openly available at https://pubmed.ncbi.nlm.nih.gov/ (accessed on 4 December 2023), https://scholar.google.com/ (accessed on 4 December 2023), https://www.embase.com (accessed on 4 December 2023), https://www.wiley.com/en-ie (accessed on 4 December 2023), and https://clinicaltrials.gov (accessed on 4 December 2023).

## References

[1] Dyrskjøt L., Hansel D.E., Efstathiou J.A., Knowles M.A., Galsky M.D., Teoh J., Theodorescu D. (2023). Bladder cancer. Nat. Rev. Dis. Primers.

[2] Fasulo V., Paciotti M., Lazzeri M., Contieri R., Casale P., Saita A., Lughezzani G., Diana P., Frego N., Avolio P. (2022). Xpert Bladder Cancer Monitor May Avoid Cystoscopies in Patients Under “Active Surveillance” for Recurrent Bladder Cancer (BIAS Project): Longitudinal Cohort Study. Front. Oncol..

[3] Contieri R., Paciotti M., Lughezzani G., Buffi N.M., Frego N., Diana P., Fasulo V., Saita A., Casale P., Lazzeri M. (2022). Long-term Follow-up and Factors Associated with Active Surveillance Failure for Patients with Non–muscle-invasive Bladder Cancer: The Bladder Cancer Italian Active Surveillance (BIAS) Experience. Eur. Urol. Oncol..

[4] Hu H., Zhou M., Yang B., Zhou S., Liu Z., Zhang J. (2022). A Systematic Review on the Role of Repeat Transurethral Resection after Initial en Bloc Resection for Non-Muscle Invasive Bladder Cancer. J. Clin. Med..

[5] Kim L.H.C., Patel M.I. (2020). Transurethral resection of bladder tumour (TURBT). Transl. Androl. Urol..

[6] Bansal A., Sankhwar S., Goel A., Kumar M., Purkait B., Aeron R. (2016). Grading of complications of transurethral resection of bladder tumor using Clavien–Dindo classification system. Indian J. Urol..

[7] Tomiyama E., Fujita K., Hashimoto M., Uemura H., Nonomura N. (2024). Urinary markers for bladder cancer diagnosis: A review of current status and future challenges. Int. J. Urol..

[8] Mossanen M., Wang Y., Szymaniak J., Tan W.S., Huynh M.J., Preston M.A., Trinh Q., Sonpavde G., Kibel A., Chang S. (2019). Evaluating the cost of surveillance for non-muscle-invasive bladder cancer: An analysis based on risk categories. World J. Urol..

[9] Petrelli F., Giannatempo P., Maccagnano C., Contieri R., Hurle R. (2021). Active surveillance for non-muscle invasive bladder cancer: A systematic review and pooled-analysis. Cancer Treat Res. Commun..

[10] Soloway M.S., Bruck D.S., Kim S.S. (2003). Expectant Management of Small, Recurrent, Noninvasive Papillary Bladder Tumors. J. Urol..

[11] Fan X., He W., Huang J. (2023). Bladder-sparing approaches for muscle invasive bladder cancer: A narrative review of current evidence and future perspectives. Transl. Androl. Urol..

[12] Contieri R., Lazzeri M., Hurle R. (2023). When and How to Perform Active Surveillance for Low-risk Non–muscle-invasive Bladder Cancer. Eur. Urol. Focus..

[13] von Deimling M., Pallauf M., Bianchi A., Laukhtina E., Karakiewicz P.I., Rink M., Shariat S., Pradere B. (2022). Active surveillance for non-muscle-invasive bladder cancer: Fallacy or opportunity?. Curr. Opin. Urol..

[14] Hurle R., Maccagnano C. (2020). Active surveillance for recurrent low-grade non-muscle-invasive bladder cancer: Can we take any advantage from the COVID-19 crisis?. Arab. J. Urol..

[15] Lozano F., Raventós C.X., Carrión A., Trilla E. (2022). Optimization biomarkers in the surveillance of non muscle invasive bladder cancer. Arch Esp. Urol..

[16] Sugeeta S.S., Sharma A., Ng K., Nayak A., Vasdev N. (2021). Biomarkers in Bladder Cancer Surveillance. Front. Surg..

[17] Harsanyi S., Novakova Z.V., Bevizova K., Danisovic L., Ziaran S. (2022). Biomarkers of Bladder Cancer: Cell-Free DNA, Epigenetic Modifications and Non-Coding RNAs. Int. J. Mol. Sci..

[18] Liberati A., Altman D.G., Tetzlaff J., Mulrow C., Gotzsche P.C., Ioannidis J.P.A., Clarke M., Devereaux P., Kleijnen J., Moher D. (2009). The PRISMA statement for reporting systematic reviews and meta-analyses of studies that evaluate healthcare interventions: Explanation and elaboration. BMJ.

[19] Holzbeierlein J.M., Bixler B.R., Buckley D.I., Chang S.S., Holmes R., James A.C., Kirkby E., McKiernan J., Schuckman A. (2024). Diagnosis and Treatment of Non-Muscle Invasive Bladder Cancer: AUA/SUO Guideline: 2024 Amendment. J. Urol..

[20] EAU Guidelines (2023). Edition presented at the EAU Annual Congress Milan 2023.

[21] Roobol M.J., Bangma C.H., el Bouazzaoui S., Franken-Raab C.G., Zwarthoff E.C. (2010). Feasibility study of screening for bladder cancer with urinary molecular markers (the BLU-P project). Urol. Oncol. Semin. Orig. Investig..

[22] Cowan B., Klein E., Jansz K., Westenfelder K., Bradford T., Peterson C., Scherr D., Karsh L., Egerdie B., Witjes A. (2021). Longitudinal follow-up and performance validation of an mRNA-based urine test (Xpert Bladder Cancer Monitor) for surveillance in patients with non-muscle-invasive bladder cancer. BJU Int..

[23] Neuzillet Y., Pradère B., Xylinas E., Allory Y., Audenet F., Loriot Y., Masson-Lecomte A., Roumiguié M., Seisen T., Traxer O. (2022). French AFU Cancer Committee Guidelines—Update 2022-2024: Non-muscle-invasive bladder cancer (NMIBC). Progrès En Urol..

[24] Babcook C.J., Goldstein R.B., Barth R.A., Damato N.M., Callen P.W., Filly R.A. (1994). Prevalence of ventriculomegaly in association with myelomeningocele: Correlation with gestational age and severity of posterior fossa deformity. Radiology.

[25] Gofrit O.N., Pode D., Lazar A., Katz R., Shapiro A. (2006). Watchful Waiting Policy in Recurrent Ta G1 Bladder Tumors. Eur. Urol..

[26] Pruthi R.S., Baldwin N., Bhalani V., Wallen E.M. (2008). Conservative Management of Low Risk Superficial Bladder Tumors. J. Urol..

[27] Hernández V., Alvarez M., de la Peña E., Amaruch N., Martín M.D., de la Morena J.M., Gómez V., Llorente C. (2009). Safety of Active Surveillance Program for Recurrent Nonmuscle-invasive Bladder Carcinoma. Urology.

[28] Hurle R., Pasini L., Lazzeri M., Colombo P., Buffi N., Lughezzani G., Casale P., Morenghi E., Peschechera R., Zandegiacomo S. (2016). Active surveillance for low-risk non-muscle-invasive bladder cancer: Mid-term results from the Bladder cancer Active Surveillance project. BJU Int..

[29] Hernández V., Llorente C., de la Peña E., Pérez-Fernández E., Guijarro A., Sola I. (2016). Long-term oncological outcomes of an active surveillance program in recurrent low grade Ta bladder cancer. Urol. Oncol. Semin. Orig. Investig..

[30] Gharibvand M., Kazemi M., Motamedfar A., Sametzadeh M., Sahraeizadeh A. (2017). The role of ultrasound in diagnosis and evaluation of bladder tumors. J. Family Med. Prim. Care..

[31] Stamatiou K., Moschouris H., Papadaki M., Perlepes G., Skolarikos A. (2011). Accuracy of modern ultrasonographic techniques in the follow up of patients with superficial bladder carcinoma. Med. Ultrason..

[32] Mukhtar S., Perry M.J.A. (2011). Future Prospects for Bladder Cancer Biomarkers. BJU Int..

[33] Chou R., Gore J.L., Buckley D., Fu R., Gustafson K., Griffin J.C., Grusing S., Selph S. (2015). Urinary Biomarkers for Diagnosis of Bladder Cancer. Ann. Intern Med..

[34] Zeng Y., Wang A., Lv W., Wang Q., Jiang S., Pan X., Wang F., Yang H., Bolund L., Lin C. (2023). Recent development of urinary biomarkers for bladder cancer diagnosis and monitoring. Clin. Transl. Discov..

[35] Castaneda P.R., Theodorescu D., Rosser C.J., Ahdoot M. (2023). Identifying novel biomarkers associated with bladder cancer treatment outcomes. Front. Oncol..

[36] Kim B., Jung M., Moon K.C., Han D., Kim K., Kim H., Yang S., Lee D., Jun H., Lee K. (2023). Quantitative proteomics identifies TUBB6 as a biomarker of muscle-invasion and poor prognosis in bladder cancer. Int. J. Cancer..

[37] Su S.-F., de Castro Abreu A.L., Chihara Y., Tsai Y., Andreu-Vieyra C., Daneshmand S., Skinner E., Jones P., Siegmund K., Liang G. (2014). A Panel of Three Markers Hyper- and Hypomethylated in Urine Sediments Accurately Predicts Bladder Cancer Recurrence. Clin. Cancer Res..

[38] Roperch J.-P., Grandchamp B., Desgrandchamps F., Mongiat-Artus P., Ravery V., Ouzaid I., Roupret M., Phe V., Ciofu C., Tubach F. (2016). Promoter hypermethylation of HS3ST2, SEPTIN9 and SLIT2 combined with FGFR3 mutations as a sensitive/specific urinary assay for diagnosis and surveillance in patients with low or high-risk non-muscle-invasive bladder cancer. BMC Cancer.

[39] Elsawy A.A., Awadalla A., Elsayed A., Abdullateef M., Abol-Enein H. (2021). Prospective Validation of Clinical Usefulness of a Novel mRNA-based Urine Test (Xpert® Bladder Cancer Monitor) for surveillance in Non Muscle Invasive Bladder Cancer. Urol. Oncol. Semin. Orig. Investig..

[40] Birkenkamp-Demtröder K., Nordentoft I., Christensen E., Høyer S., Reinert T., Vang S., Borre M., Agerbæk M., Jensen J.B., Ørntoft T.F. (2016). Genomic Alterations in Liquid Biopsies from Patients with Bladder Cancer. Eur. Urol..

[41] Xu Y., Kim Y.-H., Jeong P., Piao X.-M., Byun Y.J., Seo S.P., Kang H., Kim W., Lee J., Ryu D.H. (2019). Urinary Cell-Free DNA IQGAP3/BMP4 Ratio as a Prognostic Marker for Non–Muscle-Invasive Bladder Cancer. Clin. Genitourin. Cancer.

[42] Song Z., Zhang Q., Zhu J., Yin G., Lin L., Liang C. (2020). Identification of urinary hsa_circ_0137439 as a potential biomarker and tumor regulator of bladder cancer. Neoplasma.

[43] Zhang M., Ren B., Li Z., Niu W., Wang Y. (2017). Expression of N-Myc Downstream-Regulated Gene 2 in Bladder Cancer and Its Potential Utility as a Urinary Diagnostic Biomarker. Med. Sci. Monit..

[44] Du L., Duan W., Jiang X., Zhao L., Li J., Wang R., Yan S., Xie Y., Yan K., Wang Q. (2018). Cell-free lncRNA expression signatures in urine serve as novel non-invasive biomarkers for diagnosis and recurrence prediction of bladder cancer. J. Cell Mol. Med..

[45] Mamdouh S., Sherif H., Romeih M., Elesaily K. (2023). Urine micro-RNA signature as a potential non-invasive diagnostic biomarker in bladder cancer. Asian Pac. J. Cancer Prev..

[46] Schneider A., Borgnat S., Lang H., Régine O., Lindner V., Kassem M., Saussine C., Oudet P., Jacqmin D., Gaub M.P. (2000). Evaluation of microsatellite analysis in urine sediment for diagnosis of bladder cancer. Cancer Res..

[47] Svatek R.S., Herman M.P., Lotan Y., Casella R., Hsieh J., Sagalowsky A.I., Shariat S.F. (2006). Soluble Fas—A promising novel urinary marker for the detection of recurrent superficial bladder cancer. Cancer.

[48] Kim Y.-H., Yan C., Lee I.-S., Piao X.-M., Byun Y.J., Jeong P., Kim W., Yun S., Kim W. (2016). Value of urinary topoisomerase-IIA cell-free DNA for diagnosis of bladder cancer. Investig. Clin. Urol..

[49] Fantony J.J., Longo T.A., Gopalakrishna A., Owusu R., Lance R.S., Foo W.C., Inman B.A., Abern M.R. (2017). Urinary NID2 and TWIST1 methylation to augment conventional urine cytology for the detection of bladder cancer. Cancer Biomark..

[50] van der Heijden A.G., Mengual L., Ingelmo-Torres M., Lozano J.J., van Rijt-van de Westerlo C.C.M., Baixauli M., Geavlete B., Moldoveanud C., Ene C., Dinney C. (2018). Urine cell-based DNA methylation classifier for monitoring bladder cancer. Clin. Epigenet..

[51] Allione A., Pardini B., Viberti C., Giribaldi G., Turini S., Di Gaetano C., Guarrera S., Cordero F., Oderda M., Allasia M. (2018). MMP23B expression and protein levels in blood and urine are associated with bladder cancer. Carcinogenesis.

[52] Ecke T., Weiß S., Stephan C., Hallmann S., Arndt C., Barski D., Otto T., Gerullis H. (2018). UBC^®^ Rapid Test—A Urinary Point-of-Care (POC) Assay for Diagnosis of Bladder Cancer with a focus on Non-Muscle Invasive High-Grade Tumors: Results of a Multicenter-Study. Int. J. Mol. Sci..

[53] Hofbauer S.L., de Martino M., Lucca I., Haitel A., Susani M., Shariat S.F., Klatte T. (2018). A urinary microRNA (miR) signature for diagnosis of bladder cancer. Urol. Oncol. Semin. Orig. Investig..

[54] Hayashi Y., Fujita K., Matsuzaki K., Eich M.-L., Tomiyama E., Matsushita M., Koh Y., Nakano K., Wang C., Ishizuya Y. (2020). Clinical Significance of Hotspot Mutation Analysis of Urinary Cell-Free DNA in Urothelial Bladder Cancer. Front. Oncol..

[55] Chen X., Zhang J., Ruan W., Huang M., Wang C., Wang H., Jiang Z., Wang S., Liu Z., Liu C. (2020). Urine DNA methylation assay enables early detection and recurrence monitoring for bladder cancer. J. Clin. Investig..

[56] Ou Z., Li K., Yang T., Dai Y., Chandra M., Ning J., Wang Y., Xu R., Gao T., Xie Y. (2020). Detection of bladder cancer using urinary cell-free DNA and cellular DNA. Clin. Transl. Med..

[57] Deng L., Chao H., Deng H., Yu Z., Zhao R., Huang L., Gong Y., Zhu Y., Wang Q., Li F. (2022). A novel and sensitive DNA methylation marker for the urine-based liquid biopsies to detect bladder cancer. BMC Cancer.

[58] Hentschel A.E., Beijert I.J., Bosschieter J., Kauer P.C., Vis A.N., Lissenberg-Witte B.I., van Moorselaar J.R., Steenbergen R., Jakko A., Nieuwenhuijzen J. (2022). Bladder cancer detection in urine using DNA methylation markers: A technical and prospective preclinical validation. Clin. Epigenet..

[59] Scarpato K.R., Tyson M.D., Clark P.E. (2016). Natural biology and management of nonmuscle invasive bladder cancer. Curr. Opin. Oncol..

[60] Tilki D., Burger M., Dalbagni G., Grossman H.B., Hakenberg O.W., Palou J., Reich O., Rouprêt M., Shariat S., Zlotta A. (2011). Urine Markers for Detection and Surveillance of Non–Muscle-Invasive Bladder Cancer. Eur. Urol..

[61] Chakraborty A., Dasari S., Long W., Mohan C. (2019). Urine protein biomarkers for the detection, surveillance, and treatment response prediction of bladder cancer. Am. J. Cancer Res..

[62] Lee H.-H., Kim S.H. (2020). Review of non-invasive urinary biomarkers in bladder cancer. Transl. Cancer Res..

[63] Vanderbilt University Medical Center (2016). Observation Versus Immediate Surgery of Low Risk Bladder Cancer (NCT02700724). NCT02700724.

[64] The University of Texas Health Science Center at San Antonio (2014). Surveillance for Low and Low-Intermediate Risk Non-Muscle Invasive Bladder Cancer: A Pilot Study (NCT02298998). NCT02298998.

[65] Reyes R.M., Rios E., Barney S., Hugen C.M., Michalek J.E., Lotan Y., Messing E., Svatek R. (2021). A Randomized Feasibility Trial Comparing Surveillance Regimens for Patients with Low and Low-Intermediate Risk Non-Muscle Invasive Bladder Cancer. Bladder Cancer.

[66] Vall Hebron Insitut Recerca (2021). Active Surveillance vs in Office Fulguration for Low Grade Bladder Cancer Tumors (NCT05148728). NCT05148728.

[67] Bilim V., Kuroki H., Shirono Y., Murata M., Hiruma K., Tomita Y. (2022). Advanced Bladder Cancer: Changing the Treatment Landscape. J. Pers. Med..

[68] Karimi A., Shobeiri P., Azadnajafabad S., Masinaei M., Rezaei N., Ghanbari A., Rezaei N., Rouhifard M., Shahin S., Rashidi M. (2022). A global, regional, and national survey on burden and Quality of Care Index (QCI) of bladder cancer: The global burden of disease study 1990–2019. PLoS ONE.

[69] Fujii T., Uchiyama T., Takeda M., Shimada K. (2022). Diagnostic Strategies for Urologic Cancer Using Expression Analysis of Various Oncogenic Surveillance Molecules—From Non-Coding Small RNAs to Cancer-Specific Proteins. Appl. Sci..

[70] Shill D.K., Roobol M.J., Ehdaie B., Vickers A.J., Carlsson S.V. (2021). Active surveillance for prostate cancer. Transl. Androl. Urol..

